# New Variety of Hernia—Hernia Destitute of Peritoneal Sac

**Published:** 1842-05

**Authors:** 


*New variety of Hernia—Hernia destitute of Peritoneal Sac.* By M. Dumeaux.—The following remarkable disposition of parts was observed in the body of a man between 55 and 60 years of of age, who had a large scrotal hernia on the right side. After removing all the coverings of the tumor, the hernial sac, or what appeared to be the sac, instead of being a uniformly resisting membrane, presented very distinct muscular fasciculi, and the posterior wall of the cæcum was immediately recognized. On examination by the abdomen it was found that the cæcum, instead of occupying the iliac fossa, was placed on the abdominal wall, and covered by peritoneum on one of its surfaces only. Its adherent wall was engaged in the inguinal canal forming part of the hernial tumor. The free wall, covered with peritoneum, followed, being as it were invaginated in the former, and forming towards the abdomen a cavity or true hernial sac, in which seven or eight inches of intestine were engaged.

This singular species of hernia has never before been described. The preparation was presented to the Anatomical Society.—*B. and F. Med. Rev.* Oct. 1841, from *Annales de Chirurg.* July, 1841.

